# Vaccination

**Published:** 1871-06

**Authors:** 


### VACCINATION.

A MILITARY surgeon lately examined a large number of recruits for the army and navy, with the result that sixty boys in every hundred, who had not been vaccinated, had had the small-pox, while of those who had been vaccinated, only two in a hundred had taken it. It is utterly useless to oppose vaccination in the face of a fact like this.

It is the testimony of many eminent medical men, that in all their experience they had never seen a case where another disease had been communicated to a person by the process of vaccination. It is to be hoped that every reader will bear in mind these two facts as long as he lives, and act up to them.

